# Exposure to Phthalates Affects Calcium Handling and Intercellular Connectivity of Human Stem Cell-Derived Cardiomyocytes

**DOI:** 10.1371/journal.pone.0121927

**Published:** 2015-03-23

**Authors:** Nikki Gillum Posnack, Rabia Idrees, Hao Ding, Rafael Jaimes III, Gulnaz Stybayeva, Zaruhi Karabekian, Michael A. Laflamme, Narine Sarvazyan

**Affiliations:** 1 Department of Pharmacology & Physiology, The George Washington University, Washington, DC, United States of America; 2 Department of Electrical & Computer Engineering, The George Washington University, Washington, DC, United States of America; 3 Department of Biomedical Engineering, University of California Davis, Davis, CA, United States of America; 4 Department of Pathology, University of Washington, Seattle, WA, United States of America; Qingdao Agricultural University, CHINA

## Abstract

**Background:**

The pervasive nature of plastics has raised concerns about the impact of continuous exposure to plastic additives on human health. Of particular concern is the use of phthalates in the production of flexible polyvinyl chloride (PVC) products. Di-2-ethylhexyl-phthalate (DEHP) is a commonly used phthalate ester plasticizer that imparts flexibility and elasticity to PVC products. Recent epidemiological studies have reported correlations between urinary phthalate concentrations and cardiovascular disease, including an increased risk of high blood pressure and coronary risk. Yet, there is little direct evidence linking phthalate exposure to adverse effects in human cells, including cardiomyocytes.

**Methods and Results:**

The effect of DEHP on calcium handling was examined using monolayers of gCAMP3 human embryonic stem cell-derived cardiomyocytes, which contain an endogenous calcium sensor. Cardiomyocytes were exposed to DEHP (5 – 50 μg/mL), and calcium transients were recorded using a Zeiss confocal imaging system. DEHP exposure (24 – 72 hr) had a negative chronotropic and inotropic effect on cardiomyocytes, increased the minimum threshold voltage required for external pacing, and modified connexin-43 expression. Application of Wy-14,643 (100 μM), an agonist for the peroxisome proliferator-activated receptor alpha, did not replicate DEHP’s effects on calcium transient morphology or spontaneous beating rate.

**Conclusions:**

Phthalates can affect the normal physiology of human cardiomyocytes, including DEHP elicited perturbations in cardiac calcium handling and intercellular connectivity. Our findings call for additional studies to clarify the extent by which phthalate exposure can alter cardiac function, particularly in vulnerable patient populations who are at risk for high phthalate exposure.

## Introduction

Di(2-ethylhexyl)phthalate (DEHP) is a commonly used phthalate plasticizer employed to impart flexibility to polyvinyl chloride (PVC) products (reviewed in [1]). Human exposure to DEHP can occur through contact with consumer products, food packaging, medical devices, water, air and dust [2]. Since DEHP is hydrophobic and not covalently bound to PVC, it is highly susceptible to leaching when in contact with lipophilic fluids. Indeed, human biomonitoring studies suggest that a large proportion of the population is routinely exposed to DEHP, including both children and adults [3–5]. As such, phthalate leaching is a source of concern for human health, particularly in the medical setting where multiple medical interventions can dramatically increase a patient’s level of exposure (reviewed in [6]).

We previously reported that DEHP exposure affects conduction in neonatal rat cardiomyocytes. Specifically, delays in conduction velocity became more severe with increasing DEHP concentrations and exposure time, ultimately resulting in a loss of cardiac network synchronicity [7,8]. 72 hr exposure to DEHP [50 μg/mL] led to uncoupling between cardiomyocytes, which caused slow propagation of fractionated wavefronts [7]. This uncoupling was attributed to decreased expression of connexin-43, a transmembrane protein that facilitates electrical coupling between neighboring cells. Using rat cardiomyocytes, we also reported changes in calcium handling following DEHP exposure, including changes in the expression of calcium handling genes and an increased incidence of calcium transient doublets [8]. These data clearly showed that DEHP adversely affects cardiac function in rodent cardiomyocytes; yet, the direct applicability of these findings to humans remains to be established.

Species differences in cardiac physiology hinder direct extrapolation of rodent data to humans [9–11]. Moreover, DEHP has been reported to exert species-specific effects on metabolism [12] and gap junctions [13], which are mediated via peroxisome proliferator-activated receptors (PPARs). Importantly, at least one study observed DEHP-induced modifications in gap junction intercellular communication in rodent hepatocytes, while hamsters or primates were unaffected [13]. Considering the public health implications of a possible link between DEHP exposure and cardiac toxicity, it is critical to determine whether phthalates initiate adverse effects on cardiomyocytes of human origin. To the best of our knowledge, our study is the first to directly examine the effect of DEHP exposure on calcium handling in human stem cell-derived cardiomyocytes (hESC-CM).

## Materials and Methods

### Materials

Cy3 and Cy5 secondary antibodies were purchased from Jackson ImmunoResearch (West Grove, CA). Roswell Park Memorial Institute (RPMI), B-27 supplement, Versene, G418, and 4,6-diamidino-2-phenylindole (DAPI) were purchased from Life Technologies (Carlsbad, CA). Activin A, Bone morphogenic protein 4 (BMP4), and human FGF basic (hbFGF) were purchased from R&D systems (Minneapolis, MN). Matrigel was purchased from BD biosciences (San Jose, CA). Rockefeller University embryonic stem cell line 2 (RUES2) human embryonic stem cells (hESCs) were kindly provided by Dr. Ali Brivanlou of Rockefeller University [14,15]. All other chemicals were purchased from Sigma Aldrich (St Louis, MO), including DEHP (lot #112K3730).

### Expression of endogenous calcium sensor

RUES2 hESCs were previously modified to express a genetically encoded fluorescent calcium sensor, GCaMP3 [16,17]. Briefly, a transgene encoding the constitutive expression of GCaMP3 was inserted into the AAVS1 locus in RUES2 hESCs using zinc finger nuclease (ZFN) technology. Two targeting vectors were co-electroporated into RUES2 cells: one containing AAVS1 ZFN and a second containing a CAG promoter driving GCaMP3 expression and a PGK promoter driving neomycin resistance expression. hESCs were cultured in murine embryonic fibroblast feeder-conditioned media (MEF-CM) supplemented with 10 μM Y-27632 Rho-associated kinase inhibitor. Fluorescent colonies were expanded and selected with 40–100 μg/mL G418 for 5–10 days. RUES2 cells are an approved cell line on the NIH Human Embryonic Stem Cell Registry (#: NIHhESC-09-0013).

### Differentiation of human stem cell-derived cardiomyocytes

Differentiation of RUES2 hESCs were performed using an established monolayer protocol, which reliably produces a high yield of cardiomyocytes [18]. Briefly, undifferentiated hESCs were dispersed into single cells using Versene, and then plated in the presence of MEF-CM supplemented with 4 ng/mL hbFGF. After reaching confluency, differentiation was induced by replacing the media with RPMI supplemented with 100 ng/mL Activin A, 1:60 Matrigel, and 2% insulin-free B-27 (day 0). After 24 hr, the media was replaced with RPMI supplemented with B-27 and 10 ng/mL BMP4 (day 1–4). Thereafter, cells were cultured in RPMI supplemented with B-27, which was replaced every 2 days for an additional 20–25 days. Cardiac differentiation was identified by the appearance of spontaneous beating activity (~ day 12) via fluorescent signals with each contraction. Cardiomyocytes were exposed to control media (supplemented with 0.1% DMSO), 5–50 μg/mL DEHP (dissolved in 0.1% DMSO) or 100 μM Wy-14,643 for 24–72 hrs. Cardiomyocytes were visualized daily to monitor the appearance and beating behavior of the cell network.

### Confocal calcium imaging

Cell culture media was replaced with 37°C Tyrode’s salt solution (supplemented with 0.1% DMSO with or without 50 μg/mL DEHP or 100 μM Wy-14,643), and spontaneous beating rate recordings were collected using a Zeiss LSM 510 confocal imaging system (488 nm excitation/505-550 nm emission filters). Cells were then equilibrated at room temperature for 20 min [19], and pace-induced calcium transient recordings were measured. In the latter, the cell network was paced using a stimulation electrode (Harvard Apparatus, Holliston MA) to which monophasic 5 msec pacing pulses were applied (4V minimum threshold, Grass Stimulator). In a second set of studies, cardiomyocytes were exposed to 20 mM caffeine in the presence of 20 mM KCl to monitor total sarcoplasmic reticulum (SR) load. Confocal imaging was accomplished at a spatial/temporal resolution of ~ 650 μm /36 fps; xt line scan resolution was ~1300 μm/650 fps.

### Immunohistochemistry

Cardiomyocyte monolayers were fixed using 4% paraformaldehyde and permeabilized with 0.1% Triton. Samples were blocked with 1% bovine serum albumin and incubated overnight at 4°C with mouse sarcomeric α-actinin (1:800) or rabbit connexin-43 (1:500). Samples were incubated with secondary antibodies, anti-mouse Cy5 or anti-mouse Cy3 (1:1000), for 1 hr at room temperature. Nuclei were counterstained with DAPI (1:300). Images were acquired and analyzed with a Zeiss LSM 510 confocal imaging system using dye-specific filter settings.

### Calcium transient analysis

The following parameters were determined from calcium transient signals: amplitude (F1/F0), 50% duration time (duration from activation time to 50% relaxation time), tau/decay time constant (time for the fluorescence signal to recover 63%) and 50% upstroke time (duration from activation to 50% upstroke time). The beginning of the upstroke was defined by the initial deflection from baseline.

### Statistical Analysis

All values are expressed as mean ± SE, with p < 0.05 considered statistically significant. Mean values are expressed as a percentage of vehicle control. Statistical analyses were performed using Student’s t-test (Prism, GraphPad Software Inc., La Jolla CA). All results were computed from n = 5–25 individual experiments. Representative traces and images are shown.

## Results

### Human stem cell-derived cardiomyocytes expressing an endogenous calcium sensor

**Fig 1:**
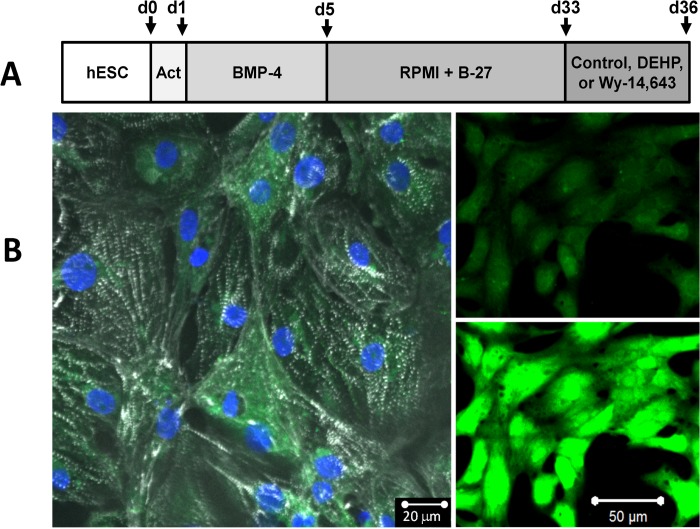
GCaMP3 expressing RUES2 hESC-CM. A) Experimental protocol for hESC differentiation to cardiomyocytes. Confluent cardiomyocyte layers were exposed to vehicle control, 5–50 μg/mL DEHP, or 100 μM Wy-14,643 for 72hrs. B) Left: Monolayers of hESC-CM stain positively for sarcomeric α-actinin (white), nuclei (blue), and GCaMP3 (green). Right: hESC-CM exhibit robust fluorescent with each contraction cycle (top—quiescent cells, bottom—calcium release).

To verify that our directed differentiation protocol (Fig. 1A) produced a highly purified cardiomyocyte population, we performed immunostaining to assess the expression of cardiac specific proteins and sarcomere organization. Immunofluorescence revealed striated staining for sarcomeric α-actinin, a protein located at the Z-line of sarcomeres in >90% of the cells (Fig. 1B). Striated labeling was observed in hESC-CMs that co-expressed the endogenous fluorescent calcium indicator, GCaMP3. GCaMP3-expressing hESC-CMs displayed robust cyclic changes in fluorescent intensity, which coincided with each contraction (Fig. 1B). hESC-CM were also loaded with Fluo-4, a exogenous calcium indicator. The outcome of the Fluo-4 experiments was identical to the gCaMP3-based studies (data not shown).

### Effect of DEHP treatment on spontaneous beating rate

**Fig 2:**
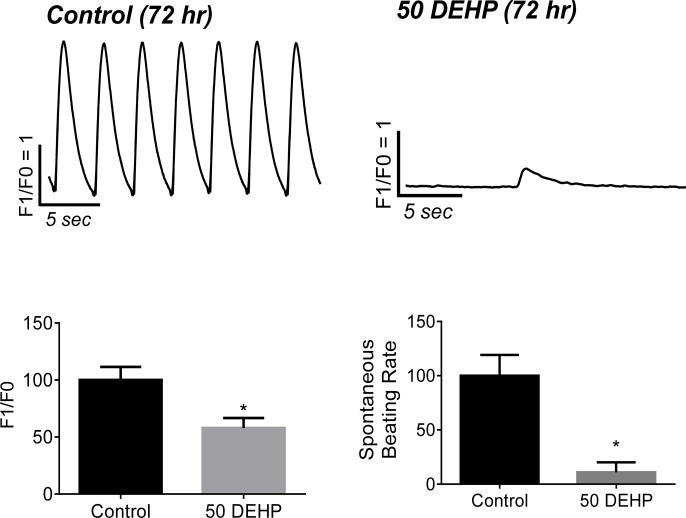
Effects of 72 hr exposure to 50 μg/mL DEHP on spontaneous calcium transients. Calcium transient recording from spontaneously beating human cardiomyocytes after 72 hr exposure to vehicle control or DEHP. DEHP treatment reduced the intrinsic beating frequency by 89% (n ≥ 9, *p* ≤ *0*.*001*), and reduced calcium transient amplitude (F1/F0) by 42% (n ≥ 9, *p* ≤ *0*.*05)* compared with control.

Individually plated hESC-CMs display variable spontaneous beating rates (SBR) that are dependent upon the specialized cell type (i.e., atrial, ventricular, nodal) [20–22]. However, when hESC-CM are grown in confluent monolayers, cells undergo phase synchronization and form an analogue of a cardiac syncytium that exhibits a more consistent, synchronized beating frequency. A number of ion channels have been shown to contribute to the balance between depolarizing and repolarizing currents in stem cell-derived cardiomyocytes [23–25]. Therefore, changes in SBR can serve as a sensitive, albeit cumulative, index of compound cardiotoxicity [26–28]. We recorded changes in the SBR of confluent, synchronously beating hESC-CM monolayers prior to treatment and again after 24–72 hr exposure to either vehicle control or DEHP-supplemented media (Fig. 1A). No significant changes in SBR were observed in vehicle control cardiomyocytes over the 72 hr time frame (data not shown, *p = 0*.*4*). However, during the same time period, DEHP exposure had a profound influence on spontaneous activity (Fig. 2), with the average SBR falling to 11% of control (*p* < 0.001). Infrequent spontaneous contractions in DEHP-treated samples were also characterized by diminished calcium transient amplitudes (F1/F0) in DEHP samples (-42%, p ≤ 0.05) compared with control samples.

### DEHP exposure perturbs intracellular calcium handling during pacing

**Fig 3:**
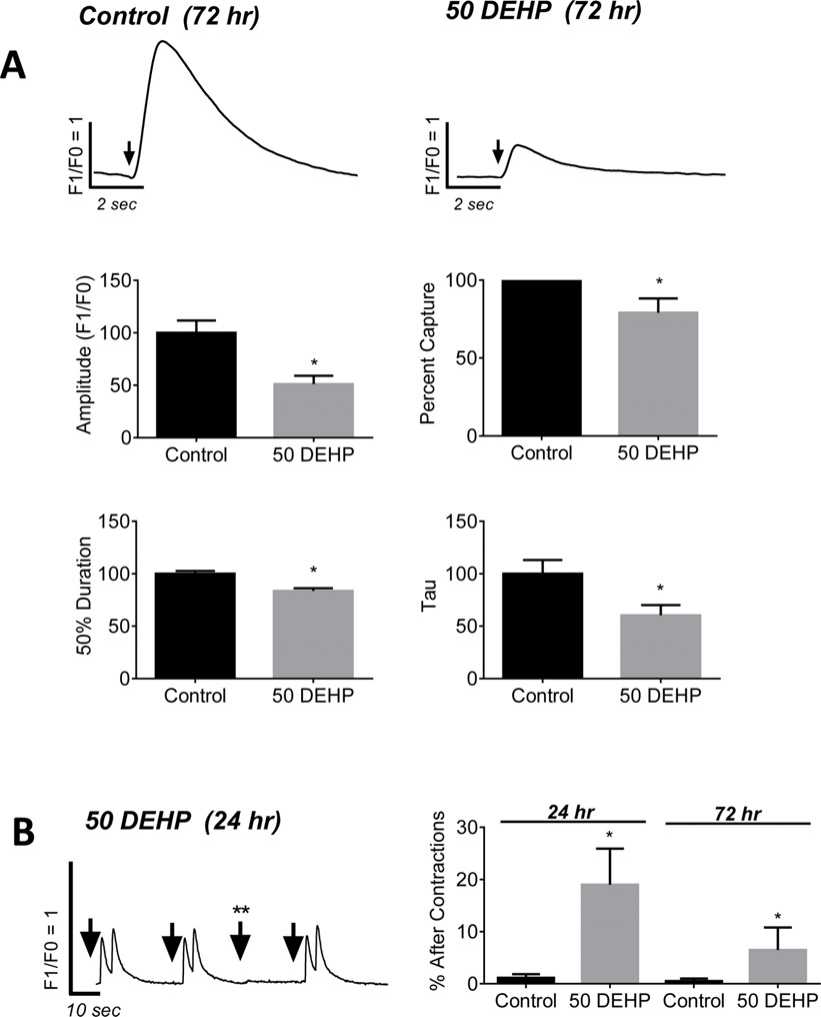
Effects of 24–72 hr exposure to 50 μg/mL DEHP on paced calcium transients. A) Action potential-driven calcium transients (↓ denotes external stimulus) from 72hr exposed control (left) or DEHP-treated cells (right). B) DEHP-treated cells display calcium transients with 49% smaller amplitudes (*p* ≤ *0*.*001*), 16% shorter duration times (*p* ≤ *0*.*001*), and 40% faster decay (*p* ≤ *0*.*01*), compared with control. No significant changes in upstroke time were observed. C) DEHP-treated cells had a greater propensity for after contractions following 24–72hrs exposure (6.5–19% of samples), and were more difficult to pace externally († stimulus does not elicit calcium transient). n ≥ 13.

Calcium transient morphology can point to changes in sarcoplasmic reticulum (SR) load and release, as well as the functional state of key calcium handling proteins such as ryanodine receptors (RyR), calcium ATPase (SERCA) and calsequestrin [29–31]. Since cardiomyocyte beating rate can have a profound effect on calcium transient morphology, including transient amplitude and duration, we investigated the impact of DEHP-treatment on paced calcium transients. hESC-CM were externally paced and the action potential-driven calcium transients were recorded and analyzed (Fig. 3A). After 72 hr exposure, calcium transient amplitudes (F1/F0 ratio) decreased by 49% in DEHP-treated cells compared with control (*p* ≤ *0*.*001*). DEHP-treated cells displayed shortened calcium transient durations (-16%, *p* ≤ *0*.*001*) and faster calcium reuptake time (-40%, *p* ≤ *0*.01). The latter can be seen as a decrease in the decay constant, tau, which is proportional to time. No significant changes in transient upstroke time were observed.

### DEHP exposure promotes aftercontractions

Triggered arrhythmias are commonly attributed to alterations in calcium handling [32–34]. Triggered arrhythmias can arise from either reactivation of calcium current caused by prolonged action potential durations, or from spontaneous SR calcium release which results in aftercontractions [33]. After 24hr exposure, 19% of DEHP-treated samples displayed after contractions in response to external pacing, compared with 1% of control monolayers (*p* ≤ *0*.*001*; Fig. 3B). This phenomenon continued throughout the 72 hr observed treatment period although to a lesser extent (6.5% of DEHP-treated vs 0.5% control, *p* ≤ *0*.*05*); the latter was primarily due to the increasing difficulty to externally pace DEHP-treated cells after 72 hr exposure, an effect that is described below in more detail.

### DEHP-treatment inhibits frequency potentiation

**Fig 4:**
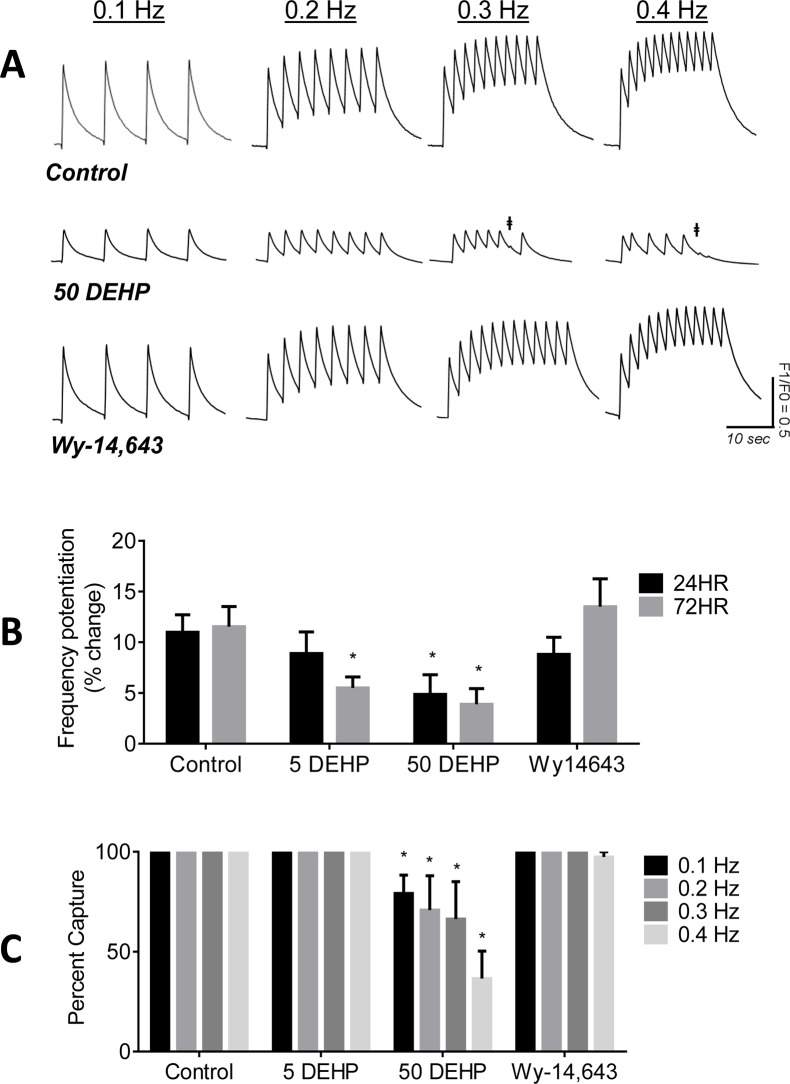
Effects of 24–72 hr exposure to 5–50 μg/mL DEHP or 100 μM Wy-14,643 on frequency potentiation. A, B) hESC-CM exhibited a positive frequency potentiation response in the presence of control (+12%), whereas DEHP-treated cells exhibited a relatively flat response (+4%). No significant differences between control and Wy-14,643 treatment were observed. C) DEHP-treated samples became increasingly more difficult to stimulate at higher pacing frequencies († stimulus does not elicit calcium transient). n ≥ 4.

Frequency potentiation is an important inotropic mechanism in normal cardiomyocytes. At faster pacing frequencies, increases in diastolic calcium and SR calcium load are observed [35]. The latter increases calcium transient amplitudes and cardiac contractility, as more calcium is available for release with the next contraction [36]. In control samples, increasing the pacing frequency resulted in an elevation of diastolic calcium levels and an increase in the amplitude of subsequent calcium transients (+12%, Fig. 4A, B). In contrast, in DEHP-treated samples, increases in pacing frequency did not augment the amplitude of calcium transients yielding a “flat response” (Fig. 4A). Moreover, higher pacing frequencies often failed to elicit calcium transients (Fig. 4A, C). At 0.4 Hz pacing frequency, 63% of DEHP-treated samples failed to capture, compared with 100% control samples.

### Effect of PPAR agonist WY-14,643 on cardiac calcium handling

Previous studies in rodent models indicated that DEHP’s toxic effects are predominately mediated by activation of PPARα, which in turn, induces peroxisome proliferation and causes hepatic toxicity[37]. Since this effect is not observed in humans, it was suggested that DEHP toxicity may be exclusive to rodents [38–40]. To decipher whether DEHP’s adverse cardiac effects were mediated by PPARα activation, hESC-CM were treated with the PPARα agonist, Wy-14,643 [7,41]. No significant effects of Wy-14,643 on frequency potentiation, or efficiency to initiate contractions with external pacing (Fig. 4A-C), calcium transient amplitude, duration or upstroke were observed (data not shown).

### Effect of DEHP on SR load

**Fig 5:**
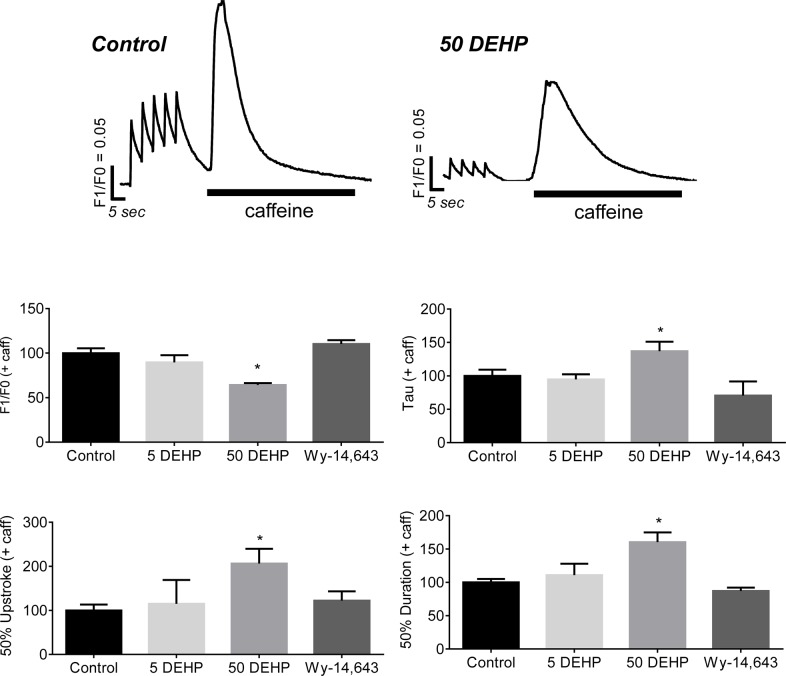
Effects of 72 hr exposure to 5–50 μg/mL DEHP or 100 μM Wy-14,643 on SR load. Top: GCaMP3 hESC-CM were paced with a train of stimuli to load the SR, and caffeine was applied to assess SR calcium load. Bottom: Caffeine-induced calcium transients were shorter (-46%) and longer (+160%) in 50 μg/mL DEHP samples compared with control. n ≥ 4.

One possible explanation for the diminished calcium transient amplitudes in DEHP-treated cells is a reduction in SR calcium load. To address this possible mechanism, hESC-CM were paced with a train of stimuli to achieve a steady state level of SR calcium load, then caffeine was applied to cell monolayers to synchronize opening of ryanodine receptors (RyR) [42]. The net result is a maximum release of calcium into the cytosol, which can be used to estimate SR calcium load. Application of caffeine produced an immediate increase in calcium transient amplitudes in both control and DEHP-treated samples. However, caffeine-induced calcium transient amplitudes were reduced by 46% and duration was increased by 160% in DEHP-treated samples compared with control (Fig. 5, *p* ≤ *0*.*01*). Lengthening of calcium transient duration time was attributed to both an increase in calcium release time (+106%, *p* ≤ *0*.*05*) and calcium reuptake time (+38%, *p* ≤ *0*.*05*). No significant differences were observed between control and Wy-14,643-treated samples.

### DEHP exposure diminishes intercellular connectivity

**Fig 6:**
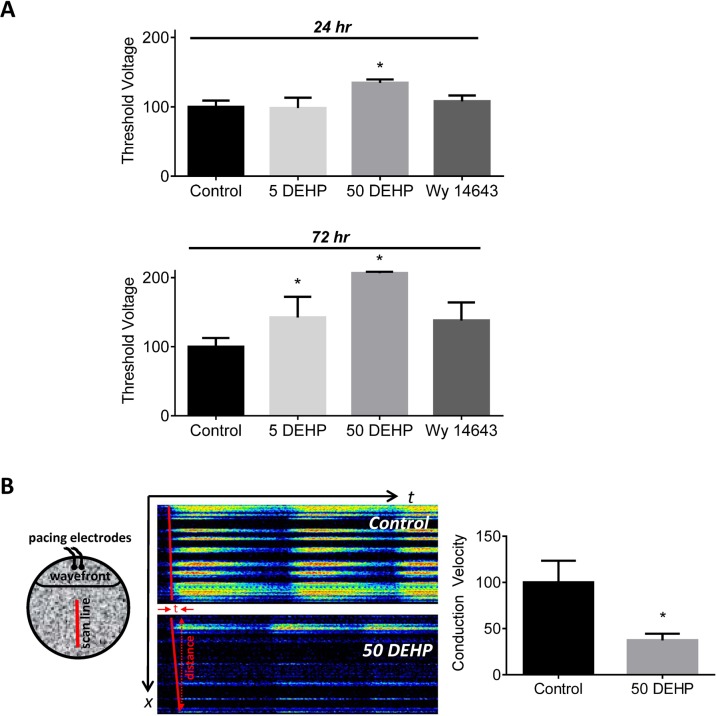
Effects of 72 hr exposure to 5–50 μg/mL DEHP or 100 μM Wy-14,643 on intercellular coupling. A) The excitation threshold voltage required to elicit a calcium transient was increased by 107% in 72 hr exposed DEHP hESC-CM monolayers, compared with control (*p* ≤ *0*.*001*; n = 6). B) Left: Cartoon illustrates xt linescan methodology across a hESC-CM cell layer. Middle & Right: xt linescan shows conduction slowing (-63%) in a monolayer of DEHP-treated cardiomyocytes; only cells near the pacing electrode (top) elicit a robust calcium transient. (*p* ≤ *0*.*05*; n = 8).

As exposure time increased, DEHP-treated cells became progressively more difficult to excite via external stimulation (Figs. 4 and 6). At 24 hr, the minimum stimulus amplitude (threshold voltage) required to consistently achieve depolarization was 35% greater in 50 μg/mL DEHP-treated cells compared with control (Fig. 6A, *p* ≤ *0*.*05*). This effect became more pronounced with time; a minimum threshold voltage was 42% greater in 5 μg/mL DEHP-treated samples (*p* ≤ *0*.*05*) and 107% greater in 50 μg/mL DEHP-treated samples (*p* ≤ *0*.*0001*) after 72 hr exposure. Deviations in threshold voltage following 100 μM Wy-14,643 72 hr treatment did not reach significance (*p = 0*.*053*). Despite increasing the stimulus voltage, propagation across the cell monolayer failed, with only cells in close proximity to the pacing electrode being excited. x-t linescan recordings indicated a 63% decrease in conduction velocity of DEHP-treated cells compared with control (*p* ≤ *0*.*05*, Fig. 6B).

### DEHP modifies connexin-43 expression

**Fig 7:**
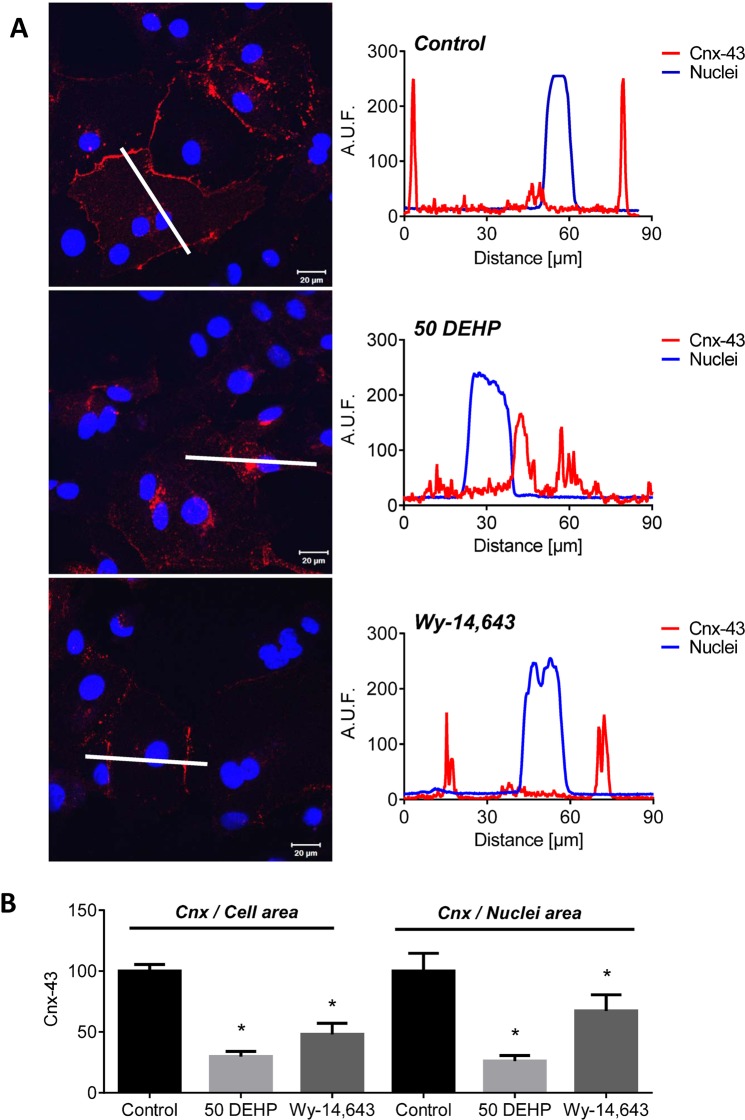
DEHP alters connexin-43 expression. A) Control cardiomyocytes display large plaques of gap junctional connexin-43 (red) on the cellular membranes; DEHP-treated cells have increased intracellular connexin-43 (red). White line denotes the region corresponding to the intensity profiles (right panel) for connexin-43 (red) and nuclear (blue) fluorescence. Wy-14,643-treated samples expressed cnx-43 on the cellular membrane, but less robustly than control. B) Total connexin-43 staining area is decreased in DEHP and Wy-14,643-treated samples—normalized to total cell area. n ≥ 4.

Gap junctions are intercellular channels that facilitate electrical communication between cardiomyocytes; we previously showed that connexin-43 (cnx-43), a protein that comprises gap junction channels, was a target of DEHP-treatment in rat cardiomyocytes [7]. Since changes in conduction velocity and excitation threshold voltage can both be attributed to diminished cell-to-cell coupling, we investigated the effect of DEHP-treatment on cnx-43 expression using immunofluorescence. In control samples, cnx-43 was intensely labeled at the cellular membrane, with large gap junctional plaques comprising a sizeable area of the cell (Fig. 7). In comparison, in DEHP-treated cells, cnx-43 was predominately perinuclear. Similar to control, Wy-14643-treated cells expressed cnx-43 largely at the plasmalemma, although less robustly. Total cnx-43 area was significantly reduced in DEHP-and Wy-14,643-treated cardiomyocytes compared with control when normalized to both total cell area (-70% and -52%, respectively) or total nuclei (-74% and -33%, respectively). No significant changes in connexin-43 gene expression was observed between control and DEHP-treated samples (*p = 0*.*2*, S1 Fig.), suggesting that DEHP’s effect on cnx-43 is not mediated by gene expression changes. This effect was previously reported for DEHP-treated rat cardiomyocytes [7].

## Discussion

DEHP is one of the most widely used phthalate plasticizers in consumer products and FDA-approved medical devices. As such, DEHP-exposure remains a public health concern, particularly for populations at risk for high exposure. The latter includes patients undergoing multiple medical procedures, such as bypass, hemodialysis or long-term use of tubing in intensive care units [1]. Since DEHP is not covalently bound to the PVC polymer and is hydrophobic, it is highly susceptible to leaching when in contact with blood, plasma, total parental nutrition solution, formulation aids used to solubilize medications, and other lipophilic fluids [43]. Exposure levels of DEHP from blood transfusion products can range from 2–83 μg/mL [44], and clinical exposure during an extracorporeal membrane oxygenation (ECMO) procedure is estimated to be 14 mg/kg/day [1]. In comparison, measured DEHP blood levels range from non-detectable to 4.71 μg/mL in normal, healthy individuals [45,46], and the median environmental DEHP exposure levels are estimated to range between 2–312 μg/kg/day [47]. The published reference dose for DEHP is 0.022 mg/kg/day, as determined by the Environmental Protection Agency [48]. Although increased phthalate exposure has been linked to a variety of adverse health outcomes in both children and adults [49–56], the impact of DEHP on human cardiac function remains largely unknown. We aimed to investigate the direct effect of DEHP on human cardiomyocytes, using clinically-relevant concentrations (50 μg/mL) and an exposure duration (24–72 hr) that is comparable to plasticizer presence in the blood of patients with high medical device usage [57,58].

We previously reported that exposure to clinically-relevant DEHP concentrations impaired electrical conduction in neonatal rat cardiomyocytes, resulting in an arrhythmogenic phenotype [7,8]. Specifically, 72 hr exposure to 50 μg/mL DEHP caused asynchronous cell beating and markedly decreased conduction velocity. These effects were mainly attributed to a loss of gap junctional connexin-43, which can impair intercellular communication [59]. Notably, DEHP, and its main metabolite MEHP, have both been reported to reduce gap junctional connections in other cell types, including testicular cells [60,61] and hepatocytes [13,62]. Importantly, a few studies have indicated species-specific outcomes related to DEHP’s effects that appear to be mediated by peroxisome proliferator receptors (PPARs). Specifically, reduced gap junctional intercellular communication was observed in rodent hepatocytes, but not in hamster, monkey or human cells [13,62]. Additionally, DEHP was shown to modify energy metabolism in rodent hepatocytes, but these effects were abolished in a humanized PPARα mouse model [12]. Of interest, we previously showed that the effects of DEHP on cardiomyocyte metabolism were only partially mimicked with a PPARα agonist [41], suggesting that these species-specific effects of DEHP may not be applicable to cardiac cells. However, the direct effect of DEHP on human cardiac cells has not been examined—and fundamental differences in cardiac physiology prevent direct extrapolation of rodent findings to humans [63].

In the present study, we examined the effect of DEHP exposure on intracellular calcium handling in hESC-CM expressing the GCaMP3 endogenous calcium sensor. Intracellular calcium is an important regulator of cardiac function, as it plays a role in cardiac electrophysiology, excitation-contraction coupling and mechanical function [33]. Indeed, this GCaMP3-expressing cell line has proven to be useful in assessing the activity of transplanted hESC-CM grafts in vivo, and stem cell coupling with the host myocardium [17]. In mature adult cardiomyocytes, calcium influx through the L-type calcium channel triggers robust calcium release from the SR via RyR during systole [33]. This calcium-induced calcium release couples electrical excitation to mechanical contraction. During diastole, calcium is removed from the cytosol via the SR calcium ATPase (SERCA) and sodium/calcium exchanger [33]. The functional characteristics of hESC-CM vary by parental cell line and maturation stage, but generally, hESC-CM exhibit greater spontaneous activity, slower conduction velocities, and less mature calcium handling properties compared with adult cardiomyocytes [30,31,64]. Our vehicle control hESC-CM displayed an SBR of 0.2 Hz and conduction velocity of 1 cm/sec, which are consistent with monitoring late-stage hESC-CM [65].

The pattern of effects observed in DEHP-treated samples (Figs. 2–7), point to three likely culprits behind the adverse effects of DEHP in hESC-CM: reduced expression and/or activity of calsequestrin (CASQ2), SR calcium ATPase (SERCA), and gap junctional cnx-43. CASQ2 is a high capacity calcium binding protein localized to the SR in the vicinity of RyR [66,67]. CASQ2 acts as an active calcium buffer that modulates local luminal calcium-dependent closure of RyRs. When intra-SR calcium levels decline, calcium free CASQ2 binds to the luminal side of RyRs, causing them to close [67,68]. Cardiomyocytes with depleted CASQ2 levels behave similar to DEHP-treated cells—lower calcium transient amplitude, faster decay time, diminished SR load and increased spontaneous triggered activity [69]. In CASQ2 null mice, reduced CASQ2 expression in nodal cells led to decreased SBR and sinoatrial bradycardia [70], similar to DEHP’s effect on hESC-CM shown here (Fig. 2). When CASQ2 levels are reduced, the functional recovery of RyR release sites is accelerated making them prone to premature or spontaneous reactivation, as we often saw in DEHP-treated cells (Fig. 3B). SERCA is a main pump that transports calcium ions from the cytoplasm into the SR. Diminished SERCA levels are believed to be the main reason for a lack of frequency potentiation in failing hearts [71]. Reduced SERCA activity can also explain longer times to reabsorb caffeine-induced calcium into the SR (Fig. 5). The latter is common after application of caffeine, which enhances SR leak, making it harder for the SR to remove calcium from the cytosol, and ultimately resulting in a decreased rate of decay of calcium transients [72]. Lastly, DEHP exposure clearly impacts the distribution of cnx-43, resulting in reduced gap junctional expression at the cell membrane. A reduction in membrane-associated cnx-43 is the most likely explanation for slowed conduction velocity and a higher activation threshold in DEHP-treated hESC-CM (Fig. 7). The adverse effects of DEHP are unlikely to be mediated exclusively through gene expression changes (S1 Fig. and S1 File); additional studies are necessary to pinpoint the mechanisms by which DEHP alters calcium handling and intercellular communication. The latter can include post-transcriptional regulation and alterations in protein expression, trafficking, stabilization and activity.

## Conclusions

Our study revealed negative chronotropic and inotropic effects of DEHP exposure, and reduced intercellular connectivity of human cardiomyocytes. Exposure to clinically-relevant DEHP concentrations reduced the spontaneous beating rate, reduced calcium transient amplitudes, shortened calcium transient duration and decreased the decay time constant. The rise and decay of calcium modulates both the contractile force and the frequency of cardiomyocyte contraction [73]. Smaller calcium transient amplitudes will result in generation of less contractile force leading to a poorer cardiac performance [33,73]. DEHP-treated cardiomyocytes also had an increased incidence of aftercontractions and reduced connexin-43 expression, suggesting that exposure to phthalates may be arrhythmogenic via a higher incidence of delayed afterdepolarization arrhythmias and slowed conduction velocity [74,75].

Our study was limited to the effects of DEHP on human cardiomyocytes, using concentrations and durations that mimic clinical exposure conditions. Oral exposure to DEHP results in rapid metabolism to MEHP and 2-ethylhexanol, however, the rate of metabolism is significantly slower upon intravenous exposure. The latter is the most common route of administration in the clinical setting (i.e., blood transfusions, hemodialysis, ECMO), which largely avoids first pass metabolism. Our previously published studies revealed adverse effects of DEHP on cardiac electrical conduction and an arrhythmogenic phenotype in rat cardiomyocytes [7]. However, additional studies are necessary to fully elucidate the exact pathways behind the adverse effects of phthalates, and their metabolites, on human cardiac muscle physiology. The latter includes investigating the potential impact of phthalates on PPAR signaling pathways in cardiac myocytes [76].

## Supporting Information

S1 Fig Quantitative real-time RT-PCR analysis. No significant changes in the gene expression of sarcoplasmic reticulum Ca2+-ATPase, muscle (SERCA2, *p = 0*.*7*), calsequestrin-2 (CASQ2, *p = 0*.*7*), ryanodine receptor 2 (RYR2, *p = 0*.*8*), and connexin-43 (cnx43, *p = 0*.*2*) were observed between control and DEHP-treated samples (n = 3).
(PDF)

S1 File Materials and methods for quantitative real-time RT-PCR. (PDF)

## References

[1] FDA (2002) Safety Assessment of Di(2-ethylhexyl)phthalate (DEHP) Released from PVC Medical Devices. Available: http://www.fda.gov/downloads/MedicalDevices/…/UCM080457.pdf.

[2] Wittassek M, Koch HM, Angerer J, Brüning T (2011) Assessing exposure to phthalates—the human biomonitoring approach. Mol Nutr Food Res 55: 7–31. 10.1002/mnfr.201000121 20564479

[3] Wittassek M, Wiesmuller GA, Koch HM, Eckard R, Dobler L, et al (2007) Internal phthalate exposure over the last two decades—a retrospective human biomonitoring study. Int J Hyg Environ Health 210: 319–333. 17400024 10.1016/j.ijheh.2007.01.037

[4] Saravanabhavan G, Guay M, Langlois É, Giroux S, Murray J, et al (2013) Biomonitoring of phthalate metabolites in the Canadian population through the Canadian Health Measures Survey (2007–2009). Int J Hyg Environ Health 216: 652–661. 10.1016/j.ijheh.2012.12.009 23419587

[5] Berman T, Hochner-Celnikier D, Calafat AM, Needham LL, Amitai Y, et al (2009) Phthalate exposure among pregnant women in Jerusalem, Israel: results of a pilot study. Environ Int 35: 353–357. 10.1016/j.envint.2008.08.010 18824263

[6] Posnack NG (2014) The Adverse Cardiac Effects of Di(2-ethylhexyl)phthalate and Bisphenol A. Cardiovasc Toxicol. 14: 339–57. 10.1007/s12012-014-9258-y 24811950 PMC4213213

[7] Gillum N, Karabekian Z, Swift LM, Brown RP, Kay MW, et al (2009) Clinically relevant concentrations of di (2-ethylhexyl) phthalate (DEHP) uncouple cardiac syncytium. Toxicol Appl Pharmacol 236: 25–38. 10.1016/j.taap.2008.12.027 19344669 PMC2670944

[8] Posnack NG, Lee NH, Brown R, Sarvazyan N (2011) Gene expression profiling of DEHP-treated cardiomyocytes reveals potential causes of phthalate arrhythmogenicity. Toxicology 279: 54–64. 10.1016/j.tox.2010.09.007 20920545 PMC3003946

[9] Rudy Y, Ackerman MJ, Bers DM, Clancy CE, Houser SR, et al (2008) Systems approach to understanding electromechanical activity in the human heart: a national heart, lung, and blood institute workshop summary. Circulation 118: 1202–1211. 10.1161/CIRCULATIONAHA.108.772715 18779456 PMC2908516

[10] Loiselle DS, Gibbs CL (1979) Species differences in cardiac energetics. Am J Physiol 237: H90–8. 464076 10.1152/ajpheart.1979.237.1.H90

[11] Bers DM (2000) Calcium fluxes involved in control of cardiac myocyte contraction. Circ Res 87: 275–281. 10948060 10.1161/01.res.87.4.275

[12] Feige JN, Gerber A, Casals-Casas C, Yang Q, Winkler C, et al (2010) The pollutant diethylhexyl phthalate regulates hepatic energy metabolism via species-specific PPARalpha-dependent mechanisms. Environ Health Perspect 118: 234–241. 10.1289/ehp.0901217 20123618 PMC2831923

[13] Isenberg JS, Kamendulis LM, Smith JH, Ackley DC, Pugh G Jr, et al (2000) Effects of Di-2-ethylhexyl phthalate (DEHP) on gap-junctional intercellular communication (GJIC), DNA synthesis, and peroxisomal beta oxidation (PBOX) in rat, mouse, and hamster liver. Toxicol Sci 56: 73–85. 10869455 10.1093/toxsci/56.1.73

[14] Rosa A, Spagnoli FM, Brivanlou AH (2009) The miR-430/427/302 family controls mesendodermal fate specification via species-specific target selection. Dev Cell 16: 517–527. 10.1016/j.devcel.2009.02.007 19386261

[15] James D, Noggle SA, Swigut T, Brivanlou AH (2006) Contribution of human embryonic stem cells to mouse blastocysts. Dev Biol 295: 90–102. 16769046 10.1016/j.ydbio.2006.03.026

[16] Chong JJH, Yang X, Don CW, Minami E, Liu Y-W, et al (2014) Human embryonic-stem-cell-derived cardiomyocytes regenerate non-human primate hearts. Nature. 510: 273–7. 10.1038/nature13233 24776797 PMC4154594

[17] Shiba Y, Fernandes S, Zhu W-Z, Filice D, Muskheli V, et al (2012) Human ES-cell-derived cardiomyocytes electrically couple and suppress arrhythmias in injured hearts. Nature 489: 322–325. 10.1038/nature11317 22864415 PMC3443324

[18] Laflamme MA, Chen KY, Naumova A V, Muskheli V, Fugate JA, et al (2007) Cardiomyocytes derived from human embryonic stem cells in pro-survival factors enhance function of infarcted rat hearts. Nat Biotechnol 25: 1015–1024. 17721512 10.1038/nbt1327

[19] Chung CS, Campbell KS (2013) Temperature and transmural region influence functional measurements in unloaded left ventricular cardiomyocytes. Physiol Rep 1: e00158 10.1002/phy2.158 24400159 PMC3871472

[20] Zhang J, Wilson GF, Soerens AG, Koonce CH, Yu J, et al (2009) Functional cardiomyocytes derived from human induced pluripotent stem cells. Circ Res 104: e30–41. 10.1161/CIRCRESAHA.108.192237 19213953 PMC2741334

[21] Ma J, Guo L, Fiene SJ, Anson BD, Thomson JA, et al (2011) High purity human-induced pluripotent stem cell-derived cardiomyocytes: electrophysiological properties of action potentials and ionic currents. Am J Physiol Heart Circ Physiol 301: H2006–17. 10.1152/ajpheart.00694.2011 21890694 PMC4116414

[22] Xu C, Police S, Hassanipour M, Li Y, Chen Y, et al (2011) Efficient generation and cryopreservation of cardiomyocytes derived from human embryonic stem cells. Regen Med 6: 53–66. 10.2217/rme.10.91 21175287 PMC3057133

[23] Van den Heuvel NHL, van Veen TAB, Lim B, Jonsson MKB (2014) Lessons from the heart: mirroring electrophysiological characteristics during cardiac development to in vitro differentiation of stem cell derived cardiomyocytes. J Mol Cell Cardiol 67: 12–25. 10.1016/j.yjmcc.2013.12.011 24370890

[24] Sartiani L, Bettiol E, Stillitano F, Mugelli A, Cerbai E, et al (2007) Developmental changes in cardiomyocytes differentiated from human embryonic stem cells: a molecular and electrophysiological approach. Stem Cells 25: 1136–1144. 17255522 10.1634/stemcells.2006-0466

[25] Zhu W-ZZ, Santana LF, Laflamme MA (2009) Local control of excitation-contraction coupling in human embryonic stem cell-derived cardiomyocytes. PLoS One 4: e5407 10.1371/journal.pone.0005407 19404384 PMC2671137

[26] Sirenko O, Cromwell EF, Crittenden C, Wignall JA, Wright FA, et al (2013) Assessment of beating parameters in human induced pluripotent stem cells enables quantitative in vitro screening for cardiotoxicity. Toxicol Appl Pharmacol 273: 500–507. 10.1016/j.taap.2013.09.017 24095675 PMC3900303

[27] Braam SR, Mummery CL (2010) Human stem cell models for predictive cardiac safety pharmacology. Stem Cell Res 4: 155–156. 10.1016/j.scr.2010.04.008 20493455

[28] Jonsson MKB, Wang Q-DD, Becker B (2011) Impedance-based detection of beating rhythm and proarrhythmic effects of compounds on stem cell-derived cardiomyocytes. Assay Drug Dev Technol 9: 589–599. 10.1089/adt.2011.0396 22085047 PMC3232633

[29] Li L-X, Chen L, Meng X-Z, Chen B-H, Chen S-Q, et al (2013) Exposure levels of environmental endocrine disruptors in mother-newborn pairs in China and their placental transfer characteristics. PLoS One 8: e62526 10.1371/journal.pone.0062526 23667484 PMC3646826

[30] Liu J, Fu JD, Siu CW, Li RA (2007) Functional sarcoplasmic reticulum for calcium handling of human embryonic stem cell-derived cardiomyocytes: insights for driven maturation. Stem Cells 25: 3038–3044. 17872499 10.1634/stemcells.2007-0549

[31] Lee Y-K, Ng K-M, Lai W-H, Chan Y-C, Lau Y-M, et al (2011) Calcium homeostasis in human induced pluripotent stem cell-derived cardiomyocytes. Stem Cell Rev 7: 976–986. 10.1007/s12015-011-9273-3 21614516 PMC3226695

[32] Bers DM (2002) Calcium and cardiac rhythms: physiological and pathophysiological. Circ Res 90: 14–17. 11786512

[33] Bers DM (2008) Calcium cycling and signaling in cardiac myocytes. Annu Rev Physiol 70: 23–49. 17988210 10.1146/annurev.physiol.70.113006.100455

[34] Pogwizd SM, Schlotthauer K, Li L, Yuan W, Bers DM (2001) Arrhythmogenesis and Contractile Dysfunction in Heart Failure : Roles of Sodium-Calcium Exchange, Inward Rectifier Potassium Current, and Residual Beta Adrenergic Responsiveness. Circ Res 88: 1159–1167. 11397782 10.1161/hh1101.091193

[35] Louch WE, Stokke MK, Sjaastad I, Christensen G, Sejersted OM (2012) No rest for the weary: diastolic calcium homeostasis in the normal and failing myocardium. Physiology (Bethesda) 27: 308–323. 10.1152/physiol.00021.2012 23026754

[36] Bers DM (2002) Cardiac excitation-contraction coupling. Nature 415: 198–205. 11805843 10.1038/415198a

[37] Klaunig JE, Babich MA, Baetcke KP, Cook JC, Corton JC, et al (2003) PPARalpha agonist-induced rodent tumors: modes of action and human relevance. Crit Rev Toxicol 33: 655–780. 14727734 10.1080/713608372

[38] Huber WW, Grasl-Kraupp B, Schulte-Hermann R (1996) Hepatocarcinogenic potential of di(2-ethylhexyl)phthalate in rodents and its implications on human risk. Crit Rev Toxicol 26: 365–481. 8817083 10.3109/10408449609048302

[39] Gonzalez FJ, Shah YM (2008) PPARalpha: mechanism of species differences and hepatocarcinogenesis of peroxisome proliferators. Toxicology 246: 2–8. 18006136 10.1016/j.tox.2007.09.030

[40] Lapinskas PJ, Brown S, Leesnitzer LM, Blanchard S, Swanson C, et al (2005) Role of PPARalpha in mediating the effects of phthalates and metabolites in the liver. Toxicology 207: 149–163. 15590130 10.1016/j.tox.2004.09.008

[41] Posnack NG, Swift LM, Kay MW, Lee NH, Sarvazyan N (2012) Phthalate Exposure Changes the Metabolic Profile of Cardiac Muscle Cells. Environ Health Perspect 120: 1243–1251. 10.1289/ehp.1205056 22672789 PMC3440133

[42] Domeier TL, Blatter LA, Zima A V (2009) Alteration of sarcoplasmic reticulum Ca2+ release termination by ryanodine receptor sensitization and in heart failure. J Physiol 587: 5197–5209. 10.1113/jphysiol.2009.177576 19736296 PMC2790258

[43] Jaeger RJ, Rubin RJ (1973) Extraction, localization, and metabolism of di-2-ethylhexyl phthalate from PVC plastic medical devices. Environ Health Perspect 3: 95–102. 4735799 10.1289/ehp.730395 PMC1474915

[44] Inoue K, Kawaguchi M, Yamanaka R, Higuchi T, Ito R, et al (2005) Evaluation and analysis of exposure levels of di(2-ethylhexyl) phthalate from blood bags. Clin Chim Acta 358: 159–166. 15893743 10.1016/j.cccn.2005.02.019

[45] Latini G, De Felice C, Presta G, Del Vecchio A, Paris I, et al (2003) Exposure to Di(2-ethylhexyl)phthalate in humans during pregnancy. A preliminary report. Biol Neonate 83: 22–24. 12566679 10.1159/000067012

[46] Latini G, De Felice C, Presta G, Del Vecchio A, Paris I, et al (2003) In utero exposure to di-(2-ethylhexyl)phthalate and duration of human pregnancy. Environ Health Perspect 111: 1783–1785. 14594632 10.1289/ehp.6202 PMC1241724

[47] Pakalin S, Aschberger K, Cosgrove O, Lund B, Paya-Perez A, et al. (2008) "Bis-(2-Ethylhexyl) Phthalate, DEHP), Summary Risk Assessment report. Available: http://publications.jrc.ec.europa.eu/repository/bitstream/111111111/1001/1/dehpsum042.pdf

[48] Environmental Protection Agency (n.d.) Di(2-ethylhexyl)phthalate (DEHP): Reference Dose. Available: http://www.epa.gov/iris/subst/0014.htm

[49] Swan SH, Main KM, Liu F, Stewart SL, Kruse RL, et al (2005) Decrease in anogenital distance among male infants with prenatal phthalate exposure. Environ Health Perspect 113: 1056–1061. 16079079 10.1289/ehp.8100 PMC1280349

[50] Hauser R, Meeker JD, Duty S, Silva MJ, Calafat AM (2006) Altered semen quality in relation to urinary concentrations of phthalate monoester and oxidative metabolites. Epidemiology 17: 682–691. 17003688 10.1097/01.ede.0000235996.89953.d7

[51] Duty SM, Singh NP, Silva MJ, Barr DB, Brock JW, et al (2003) The relationship between environmental exposures to phthalates and DNA damage in human sperm using the neutral comet assay. Environ Health Perspect 111: 1164–1169. 12842768 10.1289/ehp.5756 PMC1241569

[52] Joensen UN, Frederiksen H, Jensen MB, Lauritsen MP, Olesen IA, et al (2012) Phthalate excretion pattern and testicular function: a study of 881 healthy Danish men. Environ Health Perspect 120: 1397–1403. 10.1289/ehp.1205113 22832070 PMC3491947

[53] Colon I, Caro D, Bourdony CJ, Rosario O (2000) Identification of phthalate esters in the serum of young Puerto Rican girls with premature breast development. Environ Health Perspect 108: 895–900. 11017896 10.1289/ehp.108-2556932 PMC2556932

[54] Stahlhut RW, van Wijngaarden E, Dye TD, Cook S, Swan SH (2007) Concentrations of urinary phthalate metabolites are associated with increased waist circumference and insulin resistance in adult U.S. males. Environ Health Perspect 115: 876–882. 17589594 10.1289/ehp.9882 PMC1892109

[55] Zhang Y, Lin L, Cao Y, Chen B, Zheng L, et al (2009) Phthalate levels and low birth weight: a nested case-control study of Chinese newborns. J Pediatr 155: 500–504. 10.1016/j.jpeds.2009.04.007 19555962 PMC12151091

[56] Meeker JD, Hu H, Cantonwine DE, Lamadrid-Figueroa H, Calafat AM, et al (2009) Urinary phthalate metabolites in relation to preterm birth in Mexico city. Environ Health Perspect 117: 1587–1592. 10.1289/ehp.0800522 20019910 PMC2790514

[57] Karle VA, Short BL, Martin GR, Bulas DI, Getson PR, et al (1997) Extracorporeal membrane oxygenation exposes infants to the plasticizer, di(2-ethylhexyl)phthalate. Crit Care Med 25: 696–703. 9142038 10.1097/00003246-199704000-00023

[58] Shneider B, Schena J, Truog R, Jacobson M, Kevy S (1989) Exposure to di(2-ethylhexyl)phthalate in infants receiving extracorporeal membrane oxygenation. N Engl J Med 320: 1563 2725593 10.1056/NEJM198906083202323

[59] Gutstein DE, Morley GE, Tamaddon H, Vaidya D, Schneider MD, et al (2001) Conduction slowing and sudden arrhythmic death in mice with cardiac-restricted inactivation of connexin43. Circ Res 88: 333–339. 11179202 10.1161/01.res.88.3.333 PMC3630465

[60] Sobarzo CM, Lustig L, Ponzio R, Suescun MO, Denduchis B (2009) Effects of di(2-ethylhexyl) phthalate on gap and tight junction protein expression in the testis of prepubertal rats. Microsc Res Tech 72: 868–877. 10.1002/jemt.20741 19526522

[61] Kang KS, Lee YS, Kim HS, Kim SH (2002) DI-(2-ethylhexyl) phthalate-induced cell proliferation is involved in the inhibition of gap junctional intercellular communication and blockage of apoptosis in mouse Sertoli cells. J Toxicol Environ Heal A 65: 447–459. 11936224 10.1080/15287390252808109

[62] Kamendulis LM, Isenberg JS, Smith JH, G. P Jr, Lington AW, et al (2002) Comparative effects of phthalate monoesters on gap junctional intercellular communication and peroxisome proliferation in rodent and primate hepatocytes. J Toxicol Env Heal A 65: 569–588. 10.1080/152873902317349736 11995694

[63] Olson EN (2004) A decade of discoveries in cardiac biology. Nat Med 10: 467–474. 15122248 10.1038/nm0504-467

[64] Satin J, Itzhaki I, Rapoport S, Schroder EA, Izu L, et al (2008) Calcium Handling in Human Embryonic Stem Cell Derived Cardiomyocytes. Stem Cells. 26: 1961–72. 10.1634/stemcells.2007-0591 18483424

[65] Dolnikov K, Shilkrut M, Zeevi-Levin N, Gerecht-Nir S, Amit M, et al (2006) Functional properties of human embryonic stem cell-derived cardiomyocytes: intracellular Ca2+ handling and the role of sarcoplasmic reticulum in the contraction. Stem Cells 24: 236–245. 16322641 10.1634/stemcells.2005-0036

[66] Gyorke S, Gyorke I, Terentyev D, Viatchenko-Karpinski S, Williams SC (2004) Modulation of sarcoplasmic reticulum calcium release by calsequestrin in cardiac myocytes. Biol Res 37: 603–607. 15709687 10.4067/s0716-97602004000400014

[67] Terentyev D, Viatchenko-Karpinski S, Valdivia HH, Escobar AL, Gyorke S (2002) Luminal Ca2+ controls termination and refractory behavior of Ca2+-induced Ca2+ release in cardiac myocytes. Circ Res 91: 414–420. 12215490 10.1161/01.res.0000032490.04207.bd

[68] Radwański PB, Belevych AE, Brunello L, Carnes CA, Györke S (2013) Store-dependent deactivation: cooling the chain-reaction of myocardial calcium signaling. J Mol Cell Cardiol 58: 77–83. 10.1016/j.yjmcc.2012.10.008 23108187 PMC4068615

[69] Kalyanasundaram A, Viatchenko-Karpinski S, Belevych AE, Lacombe VA, Hwang HS, et al (2012) Functional consequences of stably expressing a mutant calsequestrin (CASQ2D307H) in the CASQ2 null background. Am J Physiol Heart Circ Physiol 302: H253–61. 10.1152/ajpheart.00578.2011 21984545 PMC3334241

[70] Glukhov A V, Kalyanasundaram A, Lou Q, Hage LT, Hansen BJ, et al (2013) Calsequestrin 2 deletion causes sinoatrial node dysfunction and atrial arrhythmias associated with altered sarcoplasmic reticulum calcium cycling and degenerative fibrosis within the mouse atrial pacemaker complex. Eur Heart J. 10.1093/eurheartj/eht452 PMC4359358 24216388

[71] Pieske B, Sütterlin M, Schmidt-Schweda S, Minami K, Meyer M, et al (1996) Diminished post-rest potentiation of contractile force in human dilated cardiomyopathy. Functional evidence for alterations in intracellular Ca2+ handling. J Clin Invest 98: 764–776. 8698869 10.1172/JCI118849 PMC507487

[72] Eisner DA, Choi HS, Diaz ME, O’Neill SC, Trafford AW (2000) Integrative Analysis of Calcium Cycling in Cardiac Muscle. Circ Res 87: 1087–1094. 11110764 10.1161/01.res.87.12.1087

[73] Li S, Chen G, Li RA (2013) Calcium signalling of human pluripotent stem cell-derived cardiomyocytes. J Physiol 591: 5279–5290. 10.1113/jphysiol.2013.256495 24018947 PMC3936367

[74] Baartscheer A, Schumacher CA, Belterman CNW, Coronel R, Fiolet JWT (2003) SR calcium handling and calcium after-transients in a rabbit model of heart failure. Cardiovasc Res 58: 99–108. 12667950 10.1016/s0008-6363(02)00854-4

[75] January CT, Fozzard HA (1988) Delayed afterdepolarizations in heart muscle: mechanisms and relevance. Pharmacol Rev 40: 219–227. 3065793

[76] Lockyer P, Schisler JC, Patterson C, Willis MS (2010) Minireview: Won’t get fooled again: the nonmetabolic roles of peroxisome proliferator-activated receptors (PPARs) in the heart. Mol Endocrinol 24: 1111–1119. 10.1210/me.2009-0374 20016041 PMC5417477

