# Supplementary material for: Exposure to Phthalates Affects Calcium Handling and Intercellular Connectivity of Human Stem Cell-Derived Cardiomyocytes
Source: PLoS One. 2015 Mar 23;10(3):e0121927. doi: 10.1371/journal.pone.0121927 (PMC4370601; doi:10.1371/journal.pone.0121927)

S1 Figure. Quantitative real-time RT-PCR analysis. No significant changes in the gene expression of sarcoplasmic reticulum Ca^{2+}-ATPase, muscle (SERCA2, $p = 0.7$), calsequestrin-2 (CASQ2, $p = 0.7$), ryanodine receptor 2 (RYR2, $p = 0.8$), and connexin-43 (cnx43, $p = 0.2$) were observed between control and DEHP-treated samples (n = 3).

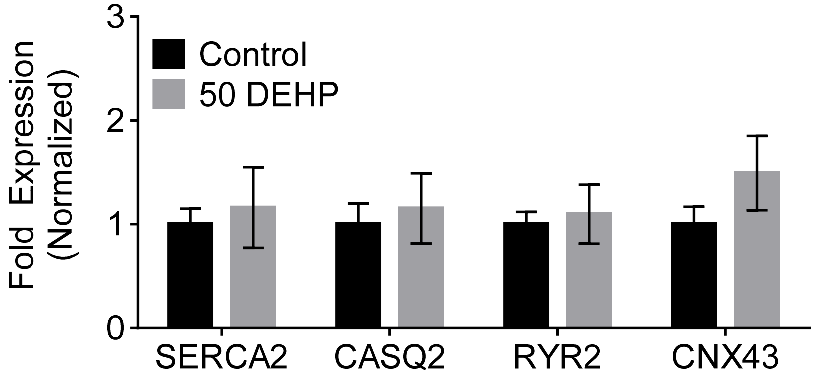

Supplement: S1 Fig — No significant changes in the gene expression of sarcoplasmic reticulum Ca2+-ATPase, muscle (SERCA2, p = 0.7), calsequestrin-2 (CASQ2, p = 0.7), ryanodine receptor 2 (RYR2, p = 0.8), and connexin-43 (cnx43, p = 0.2) were observed between control and DEHP-treated samples (n = 3). (PDF) [file pone.0121927.s001.pdf]
