# Supplementary material for: Exposure to Phthalates Affects Calcium Handling and Intercellular Connectivity of Human Stem Cell-Derived Cardiomyocytes
Source: PLoS One. 2015 Mar 23;10(3):e0121927. doi: 10.1371/journal.pone.0121927 (PMC4370601; doi:10.1371/journal.pone.0121927)
Supplement: S1 File — (PDF) [file pone.0121927.s002.pdf]

S1 File: Materials and methods for quantitative real-time RT-PCR (S1 Figure).

hESC-CM were treated for 3-days with control (0.1% DMSO) or DEHP (50 μg/mL); thereafter, RNA was isolated from cell preparations using an RNeasy kit (Qiagen, Germantown MD), per the manufacturer's instructions. Total RNA was reverse transcribed using an AffinityScript QPCR cDNA synthesis kit (Agilent Technologies, Santa Clara CA). The resulting cDNA was used as a template for quantitative real-time RT-PCR (qRT-PCR) using Taqman Gene Expression assays (Life Technologies, Carlsbad CA) for glyceraldehyde 3-phosphate dehydrogenase (GAPDH), sarcoplasmic reticulum Ca^{2+}-ATPase, muscle (SERCA2), calsequestrin-2 (CASQ2), ryanodine receptor 2 (RYR2), and connexin-43 (cnx43). qRT-PCR was performed using a CFX384 detection system (Bio-Rad, Hercules CA). Quantification and normalization of relative gene expression was accomplished using the comparative CT method (ΔΔCT). ΔΔCT values were converted to ratios by $2^{-\Delta\Delta CT}$ averaged across replicates. The expression of GAPDH was used for normalization.
